# There is one more thing to be done: ECMO!

**DOI:** 10.5935/1678-9741.20150066

**Published:** 2015

**Authors:** Fernando Antoniali

**Affiliations:** 1 Coordinator of the Pediatric Cardiac Surgery at PUC-Campinas and the Cardio Surgical Clinic ECMO group of Campinas linked to ELSO, Campinas, SP, Brazil.

*" – Unfortunately, it is not possible to get out of cardiopulmonary bypass. We have already tried several times and it is not working. We will have to let the patient die! I will talk to the family..."*

This is an extremely distressing situation for all the team involved in a cardiac surgery. Everyone who has been present in a moment like this and have the humility to recognize certainly will not deny how difficult it is to make the decision: turn off the pump and allow the patient to die. Especially, if the surgery is going on for several hours and there were many attempts to wean from cardiopulmonary bypass (CPB). Especially, if the patient is a child and the parents are outside waiting anxiously for a successful surgical repair.

However, for the most part of these patients, we can say that there is one more thing to be done. We cannot use this claim for all the patients with cardiac and respiratory failure after a heart surgery but for the most part of them, we can! For sure! Despite some of these patients can get out of the operating room with high doses of vasoactive drugs or high parameters of mechanical ventilation, the circulatory failure and severe hypoxia will culminate with important acidosis, multiple organ failure and the patient will die a few days later. The post-cardiotomy ECMO (Extracorporeal Membrane Oxygenation) is the best option to support these patients. Nevertheless, it is necessary to use a real post-cardiotomy ECMO because keeping the patient on CPB in the ICU will not work.

If we are talking about ECMO, the numbers from the ELSO registry (Extracorporeal Life Support Organization) must be highlighted. The last ELSO international report was on July 2015 and there were 69.114 ECMO patients in the registry with an overall survival of 59%^[1]^. Inside of this big group of patients, 10.183 cases were neonates and infants under 16 years old with congenital heart problems and they were supported with a cardiac ECMO. The majority of them received the support because of cardiac failure after CPB or during the first postoperative day. In this post-cardiotomy ECMO group, the mean survival was 42.7%.

Using these information from ELSO, at least we should say that it is mandatory remember about ECMO as a possibility of treatment for a patient with cardiac failure after CPB. However, it is not a widespread knowledge in our country and some people don't understand it. There is no doubt that the treatment with ECMO is increasing in Brazil but we still have to expand the information about it and improve the quality of this therapeutic technique in our country. Another important action would be the incorporation of this technology on the treatment of our patients from the public health system (SUS).

In order to spread information about ECMO, the First Latin American ELSO Conference was performed in Brazil on December 2014. There were over 500 health professionals present with the majority of Brazilians and this event was the first scientific meeting of the Latin American chapter of ELSO that was created in 2012 following examples as the Euro ELSO and Asian-Pacific ELSO. The registrants discussed different issues about cardiopulmonary support with more than 20 international speakers, including Dr. Robert Bartlett, called "The Father of ECMO" who, on the opening ceremony, talked about the experimental studies of his group in the 1960's and also about the first ECMO survival patients in the 1970's. Fortunately, ECMO is not an experimental therapy anymore and the fact of more than 69 thousands of patients had been treated until now can prove it.

READ ARTICLE ON PAGE 409

The article "Post-cardiotomy ECMO in pediatric and congenital heart surgery: impact of team training and equipment in the results" published in this issue by Leonardo Miana and all the group of INCOR-SP (see page 409), demonstrated that 0.5% of the pediatric patients operated with the use of CPB needed ECMO because of cardiac or pulmonary failure. This information is in agreement with the numbers related for other groups, whose mention between 0.5 and 2% for adults and infants. Therefore it is not a rare situation and we need to pay attention. Everyone doing more than 10 patients a month probably will have a case to use post-cardiotomy ECMO. However, if there is no concern about the quality of this type of treatment, the survival rate will be very low and spending money, time and energy of the team, will not be worth. On a scenario when very few patients survive and go home, ECMO will be called as "**E**ste **C**liente **M**orreu **O**ntem" that means in English: "This patient died yesterday" - or even worse "**E**sta **C**riança **M**orreu **O**ntem", when we are talking about pediatric patients: "This child died yesterday".

The INCOR-SP group points out the changes on the results after incorporating new materials and equipments and in special after staff training. The statistical difference between the survival rate of 5% before the new ECMO program and 45% nowadays is evident and confirms that it is the right way. The good news is that we already have in Brazil almost all the adequate materials and equipments to do ECMO in a high level. And so, we should worry with staff training. It is necessary a change of concepts! ECMO is not a treatment in which the responsible professionals are the cardiac surgeon and the perfusionist. The participation of the intensivists and the nursing staff is essential. In fact, the concept of an "ECMO specialist" is the ELSO proposal and it includes in the same group, the perfusionists, the nurses and even the respiratory therapists, which - after training - would have the ability to take care of the ECMO circuit and components, checking the quality of them, collecting samples for laboratory exams, administering volumes and drugs, changing parameters and even solving possible complications. This training program has to be done with classroom instructions and practical simulations, and must be repeated periodically because the number of cases can be small and the team has to be always prepared for the next one.

The results also improved in Campinas-SP since we put in practice this "change of concepts" at the hospitals which our team – the Clínica Cardio Cirúrgica Campinas – is working. The participation of the intensive physicians and the nursing staff, taking care of the ECMO patients, makes that the maintenance of the medical management with ECMO becomes a group decision and not only a surgeon procedure. Specially, on the pediatric group, the information about a possible post-cardiotomy ECMO is previously communicated to the ICU team and it reduces the pressure on the surgical team. Moreover, as our team works in general hospitals, the good results with the ECMO support changed the mind of the ICU groups and nowadays we are having neonates, infants and adults patients, going on ECMO for exclusively respiratory problems. In conclusion, we can mention our results to strengthen the concept that training is the right way to go because our ECMO weaning rate has increased from 60% to 88.9% and our late survival, after hospital discharge, from 10% to 77.8% in the pediatric group (cardiac and respiratory patients)^[2]^.

It is necessary an "ECMO team" with surgeons, intensivists and "ECMO specialists" working together in a reference center. In fact, ECMO must not be done at all hospitals in the country because it would increase the costs and certainly the results would be poor. However, the centers prepared for that must receive payment for all the staff involved 24 hours a day and for the materials and equipments used to keep the patients on ECMO support. The CESAR trial compared adults patients with ARDS treated with optimized mechanical ventilation in a group and on ECMO support in the other. The mortality (37% X 53%) and the costs were lower in the ECMO group. One important aspect of this UK study was that the patients on ECMO were treated in reference centers^[3]^.

Before ending this editorial, I would like to talk about a wrong decision made by CONITEC – "Comissão Nacional de Incorporação de Tecnologias no SUS". Recently, this committee didn't approve the request to incorporate ECMO support for patients of our public health system ("Sistema Único de Saúde" - SUS)^[4]^. This request was made based in a detailed technical and scientific advice ("Parecer Técnico Científico" – PTC) which was extremely accepted in an open public consultation. Surprisingly, the CONITEC's decision was in the opposite direction compared to what is happening in Brazil. The use of ECMO has increased and the results are improving mostly because of the training courses which are been offered in different parts of our country. After the First Latin American ELSO Conference, the number of Brazilians centers linked to ELSO, following the guidelines and reporting their numbers to the same registry, grew up from 3 to 9! In addition, there are others centers doing ECMO in Brazil and they intend to be part of ELSO too! Probably, very soon we will have more Brazilians scientific articles as the one published in this issue of BJCVS. Recently, Lima et al.^[5]^ demonstrated the advantages with the ECMO support to save patients after Heart Transplants in Brasília – capital city of Brazil. Another interesting paper about the economic aspects of ECMO to adult patients with ARDS in Brazil, was written by Park et al.^[6]^ and it informs that this technology is cost effective.

And even though the number of Brazilians papers about ECMO is too small at this time – we can find just 25 in the MEDLINE with the terms "ECMO" and "Brazil" – there are over 8.000 international papers about ECMO in the same bibliographic database and less than 150 are experimental studies. And even more, among this huge number of scientific articles, 1.750 are reviews! There are many studies discussing the increasing rates of survival but if we look on the numbers of the last ELSO report, the survival rates for cardiac ECMO are 41%, 51% and 42% for neonates, pediatrics and adults, respectively and talking about respiratory ECMO the numbers are even better with 74%, 57% and 58% of survival for the same groups^[1]^. Changing these percentages for absolute numbers we have 38.616 patients that survived with ECMO. Therefore there is no reason to affirm that such a treatment which has saved more than 38 thousands of lives is not good for our public health system (SUS) patients!

In conclusion, there are still some actions to be done regarding the use of ECMO in our country. We need to reverse the CONITEC decision. We need to have more ECMO support for patients with cardiopulmonary failure after CPB. We need to train people and we need to have reference centers which can receive patients to be ECMO supported with good results. We need to use ECMO to save more lives!

Really, there is one more thing to be done: ECMO!

And so, we may use another approach for difficult situations that certainly we will need to face:

"- *Unfortunately, we are not doing a good job trying to wean from CPB. I prefer not to overdo it with high levels of vasoactive drugs and bad ventilation. We had already planned and everyone is advised. Let's take the patient to the ICU on ECMO*."
